# Genome Sequence for Shiga Toxin-Producing *Escherichia coli* O26:H11, Associated with a Cluster of Hemolytic-Uremic Syndrome Cases in South Africa, 2017

**DOI:** 10.1128/genomeA.00989-17

**Published:** 2017-09-21

**Authors:** Anthony M. Smith, Kalule J. Bosco, Mark P. Nicol, Jackie Kleynhans, Mignon McCulloch, Sanelisiwe T. Duze, Arshad Ismail, Mushal Allam, Nomsa P. Tau, Karen H. Keddy

**Affiliations:** a Centre for Enteric Diseases, National Institute for Communicable Diseases, National Health Laboratory Service, Johannesburg, South Africa; b Faculty of Health Sciences, University of the Witwatersrand, Johannesburg, South Africa; c Division of Medical Microbiology, Faculty of Health Sciences, University of Cape Town, Cape Town, South Africa; d National Health Laboratory Service, Cape Town, South Africa; e South African Field Epidemiology Programme, National Institute for Communicable Diseases, National Health Laboratory Service, Johannesburg, South Africa; f Red Cross Children’s Hospital, University of Cape Town, Cape Town, South Africa; g Sequencing Core Facility, National Institute for Communicable Diseases, National Health Laboratory Service, Johannesburg, South Africa

## Abstract

Shiga toxin-producing *Escherichia coli* (STEC) strains are primarily foodborne pathogens that may cause diarrheal outbreaks and are associated with severe complications, specifically hemolytic-uremic syndrome (HUS). We report here genome sequence data for STEC O26:H11, which is associated with a cluster of cases of HUS, a rarely described syndrome in South Africa.

## GENOME ANNOUNCEMENT

Shiga toxin-producing *Escherichia coli* (STEC) strains are primarily foodborne pathogens that may cause diarrheal outbreaks and are associated with severe complications, specifically hemolytic-uremic syndrome (HUS) (1–3). STEC strains belonging to serogroups O26, O45, O103, O111, O121, and O145 are collectively referred to as the “big six” globally emerging non-O157 STEC strains (4, 5). Within the big six, serogroup O26 STEC strains are most commonly recognized (6). Very few published data exist concerning the prevalence and epidemiology of STEC infections in humans in southern Africa, where the disease is rarely reported (7). To prevent, investigate, and control outbreaks of disease, it is vital to have information about the molecular epidemiology of the disease-causing pathogen. In particular, genome sequence data can be used to investigate the population structure and evolution of pathogens. Here, we describe genome sequence data for an isolate of STEC O26:H11 associated with a cluster of HUS cases in South Africa, 2017.

In 2017, a cluster of four HUS cases that were linked temporally and geographically in South Africa was investigated. All patients were female and between the ages of 8 months and 5 years. Delayed laboratory testing of stool specimens affected the outcome of the culture and identification of bacterial pathogens. For one case, laboratory testing of a stool specimen cultured and identified a STEC O26 strain. For a second case, the stool was PCR positive for the *stx2* gene (a marker for STEC). For the single-cultured STEC isolate, analysis of genome sequence data using multiple online analysis tools (pipelines) available at the Center for Genomic Epidemiology (CGE) of the Technical University of Denmark (http://www.genomicepidemiology.org) further characterized the STEC isolate as follows: serotype O26:H11; presence of *eae*, *stx2a*, and *stx2b* virulence genes; multilocus sequence type 21; and absence of acquired antimicrobial resistance genes.

For whole-genome sequencing, genomic DNA was isolated from bacteria using the Qiagen QIAamp DNA minikit (Qiagen, Hilden, Germany). DNA libraries were prepared using a Nextera XT DNA library preparation kit (Illumina, San Diego, CA, USA), followed by 2 × 300 paired-end sequencing runs with 100× coverage using Illumina MiSeq equipment. The paired-end reads were quality trimmed using CLC Genomics Workbench version 10 software (Qiagen) and *de novo* assembled using SPAdes software (8). The assembly produced 283 contiguous (contig) sequences of longer than 500 bp, with an *N*_50_ contig value of 89,060 bp and a longest contig size of 246,873 bp. Contig measurements covered a genome size of 5,532,869 bp with a G+C nucleotide content of 50.63%. The NCBI Prokaryotic Genome Annotation Pipeline (9) determined the presence of 5,605 protein-coding genes, 385 pseudogenes, and 124 RNA genes.

### Accession number(s).

This whole-genome shotgun project has been deposited at DDBJ/ENA/GenBank under the accession number NGBP00000000. The version described in this paper is the second version, NGBP02000000.
